# Exploring patterns of accelerometry-assessed physical activity in elderly people

**DOI:** 10.1186/1479-5868-11-28

**Published:** 2014-02-28

**Authors:** Sandra Ortlieb, André Dias, Lukas Gorzelniak, Dennis Nowak, Stefan Karrasch, Annette Peters, Klaus A Kuhn, Alexander Horsch, Holger Schulz

**Affiliations:** 1 Institute of Epidemiology I, Helmholtz Zentrum München, German Research Center for Environmental Health, Neuherberg, Germany; 2 Institute of Medical Statistics and Epidemiology, TUM, Munich, Germany; 3 Computer Science Department, University of Tromsø, Tromsø, Norway; 4 Tromsø Telemedicine Laboratory, Norwegian Center for Integrated Care and Telemedicine, Tromsø, Norway; 5 Institute and Outpatient Clinic for Occupational, Social and Environmental Medicine, University Hospital of Munich (LMU), Munich, Germany; 6 Comprehensive Pneumology Center Munich (CPC-M), German Center for Lung Research, Munich, Germany; 7 Institute and Outpatient Clinic for Occupational, Social and Environmental Medicine, Ludwig-Maximilians-Universität, Munich, Germany; 8 Institute of General Practice, University Hospital Klinikum rechts der Isar, Technische Universität München, Munich, Germany; 9 Institute of Epidemiology II, Helmholtz Zentrum München, German Research Center for Environmental Health, Neuherberg, Germany; 10 Department of Clinical Medicine, University of Tromsø, Tromsø, Norway; 11 Institute of Epidemiology I, Helmholtz Zentrum München, German Research Center for Environmental Health, Ingolstädter Landstrasse 1, 85764 Neuherberg, Germany

**Keywords:** Elderly, Aged, Older, Physical activity, Exercise, Pattern, Intensity, Movement, Ambulation

## Abstract

**Background:**

Elderly people obtain significant health benefits from physical activity (PA), but the role of activity patterns has scarcely been researched. The present study aims to describe the patterns of PA among different intensities of activity in elderly people. We assess how patterns differ between more and less active groups (‘rare’, ‘average’, and ‘frequent’), and explore whether and how various PA parameters are associated with functional exercise capacity (FEC).

**Methods:**

PA was measured in 168 subjects (78 males; 65–89 years of age), using a triaxial GT3X accelerometer for ten consecutive days. Subjects were divided into three groups by activity and the groups were compared. A multiple linear regression model was used to predict FEC.

**Results:**

Participants greater than or equal to 80 years are most prone to being sedentary for long periods, while women and the obese are the groups most likely to spend insufficient time in moderate to vigorous PA (MVPA). Rarely active elderly people had a decreased proportion of long bouts of MVPA and light PA and of short bouts in sedentary behavior than frequently active subjects did (p < 0.001). As predictors of FEC, younger age, lower BMI, male sex, better lung function, absence of multimorbidity, longer times and longer bouts of MVPA emerged as significant parameters (r^2^ = 0.54). Patterns of MVPA explained most of the variance.

**Conclusions:**

PA patterns provide information beyond reports of activity alone. MVPA in elderly people may be increased by increasing the proportion of long bouts, in order to increase FEC as well as average PA. However, health conditions may limit PA. In rarely active people (often with reduced FEC, worse lung function, and diagnosis of multimorbidity or disability), longer periods of time in light PA may be sufficient to increase the overall level of activity.

## Background

Physical activity (PA) in elderly people helps maintain health, independence and quality of life, and diminishes the burden on health and social care [1]. In 2010 the World Health Organization (WHO) stated that adults of all ages should perform a minimum of 150 minutes of moderate to vigorous PA (MVPA) per week in bouts of at least 10 minutes [2]. This is more than most of the older adults achieve. In a large study by Tucker et al. [3], only 47% to 63% of US people aged 60 or older met the US guidelines for PA based on self-report: based on objectively measured PA, only 6% to 26% of elderly adults met the guidelines. Although estimates of PA vary, there is a clear need to promote PA among elderly people.

Accelerometers, which record movement over a certain period of time, are the most common instrument for the objective monitoring of PA in large epidemiological studies. The output is usually expressed as ‘activity counts’, which characterize the duration and intensity of movement of the accelerometer and thus the subject. Several studies use these signals to characterize the time or proportion of time per day spent in different intensity levels of activity. Few studies describe patterns of PA, such as frequency and duration of PA bouts or intervals in different intensity levels: even fewer look specifically at older adults [4].

Activity patterns have been proposed as a new group of PA outcomes, which may offer additional information beyond reports of activity counts and activity type recognition [4]. In 2007, a method was developed to quantify the number of activity epochs in a day and to estimate random minute-to-minute fluctuations in activity [5]. The study assessed age-related trends in adults and showed that the new measures to describe PA pattern were more sensitive than mean parameters of activity, suggesting a shift with age towards less complex, less physiologically demanding patterns of activity. Chastin and colleagues [6] extended the work by using the ‘GINI-index’ (G), a measure of inequality popularized in economic literature to measure inequality of bout lengths. This describes the pattern of accumulation of sedentary time and thus enables to evaluate and quantify sedentary behavior. By extension, this index can be applied to light, moderate and vigorous activity as well.

Recent evidence suggests that the interaction between periods of sedentary and active behavior provides different health information than the assessment of mean activity parameters alone: thus many studies focus on the pattern of sedentary behavior and walking [5-7]. However, as “aerobic activity should be performed in bouts of at least 10 minutes duration” to achieve beneficial health effects [2], it is essential to reflect the patterns of MVPA as well. Consequently, we aim to describe the patterns of PA in all three intensity levels: sedentary, light, and MVPA.

We compare the accumulated time in the intensity levels (sedentary PA, light PA, MVPA) among elderly people with different levels of activity as well as the activity patterns between these groups (‘rare’, ‘average’, and ‘frequent’). Subjects’ age, gender, and body mass index (BMI) are used to compare different PA parameters. We identify different risk groups and provide recommendations for behavioral interventions to support prevention of inactivity and related diseases in elderly people.

Furthermore, we examine the associations between several PA parameters and the clinical, well-established functional exercise capacity test (6 minutes walking test, 6MWT) [8], in order to assess the role of accumulated time spent in different intensity levels and related PA patterns in predicting functional exercise capacity.

## Methods

### Study population

Participants were a subsample of the ‘Kooperative Gesundheitsforschung in der Region Augsburg’ (KORA) study [8]. The KORA-Age study was approved by the ethics committee of the State Board of Physicians, written informed consent has been obtained from the participants and all investigations have been conducted according to the principles expressed in the Declaration of Helsinki.

200 subjects were extracted from the first and fourth quartiles of lung function from the study population and grouped by ‘better’ or ‘worse’ lung function. Nine subjects refused to attend due to personal or organizational reasons. PA levels from the non-dominant side of the hip were assessed by means of a GT3X (ActiGraph, Pensacola, FL, USA) accelerometer in 191 elderly subjects over 10 days during everyday life. More detailed information about the methods is presented in Additional file 1.

### Wear time calculation

PA data were downloaded using the ActiLife Software 4.0 (ActiGraph) and were further processed using MATLAB R2012a (MathWorks, Natick, MA, USA). We applied an adjusted algorithm of Hecht et al. [9] to determine the wearing time. PA data from the first recorded day were eliminated. Furthermore, subjects were excluded if they did not reach a minimum of four valid days (≥ 10 hours of recording/day). 168 subjects (88.9%) were eligible for analysis.

### Data processing and accelerometer measures

**Figure 1:**
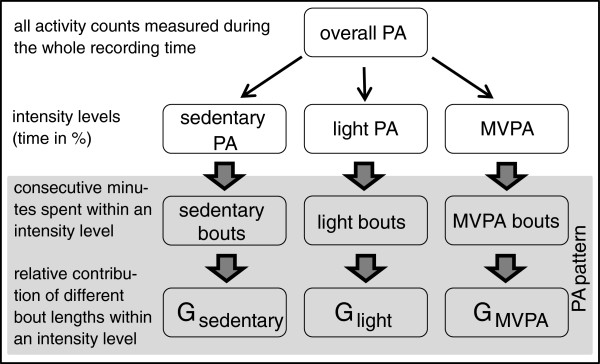
**Structure of physical activity variables.** PA = physical activity; G = GINI-Index; *high G* = mainly few long bouts are responsible for the activity pattern; *low G* = mainly short bouts of similar length contribute to the activity pattern.

Several variables were obtained from accelerometer data (uniaxial, 60-second epochs) to represent the characteristics of PA. Figure 1 presents an overview of the most important PA variables concerning this analysis. Parameters used in this study were:

#### Average activity

Average activity is the total number of counts for all valid days, divided by wearing time, for each individual. This variable was divided into 3 groups: ‘rare’ (< 25th percentile), ‘average’ (≥ 25th – < 75th percentile) and ‘frequent’ (≥ 75th percentile).

#### Intensity levels

**Figure 2:**
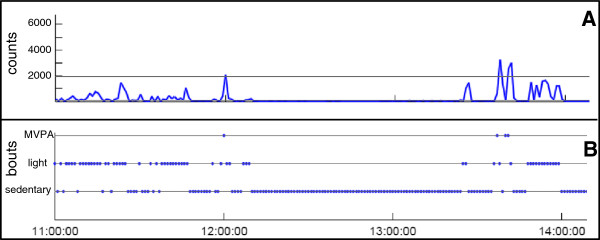
**Visualization of PA counts per minute (A) and PA bouts (B) over three hours.** PA = physical activity; MVPA = moderate to vigorous physical activity. **A)** The two lines (at 100 and 1952 counts) reflect the cut-points for light activity and MVPA. Values ≤ 100 correspond to sedentary PA, values between 100–1951 to light PA and ≥ 1952 to MVPA [10]. **B)** 1-minute-bout.

Activity counts were assigned to the different intensity levels using cut points published by Freedson et al. [10] for light, moderate, and vigorous PA (Figure 2A). Sedentary behavior was classified as ≤ 100 counts per minute.

We used the Freedson kcal equation, which provides most comparable data [11] and gives close estimates of both light and moderate PA [12]. We present the results of MVPA based on both equations in the additional file (see Additional file 2: Table S1) in order to compare Freedson’s cut points with the more recently developed cut points by Copeland and Esliger for elderly people [13]. This indicates how the choice of cut points may influence estimation of PA.

#### PA patterns

**Figure 3:**
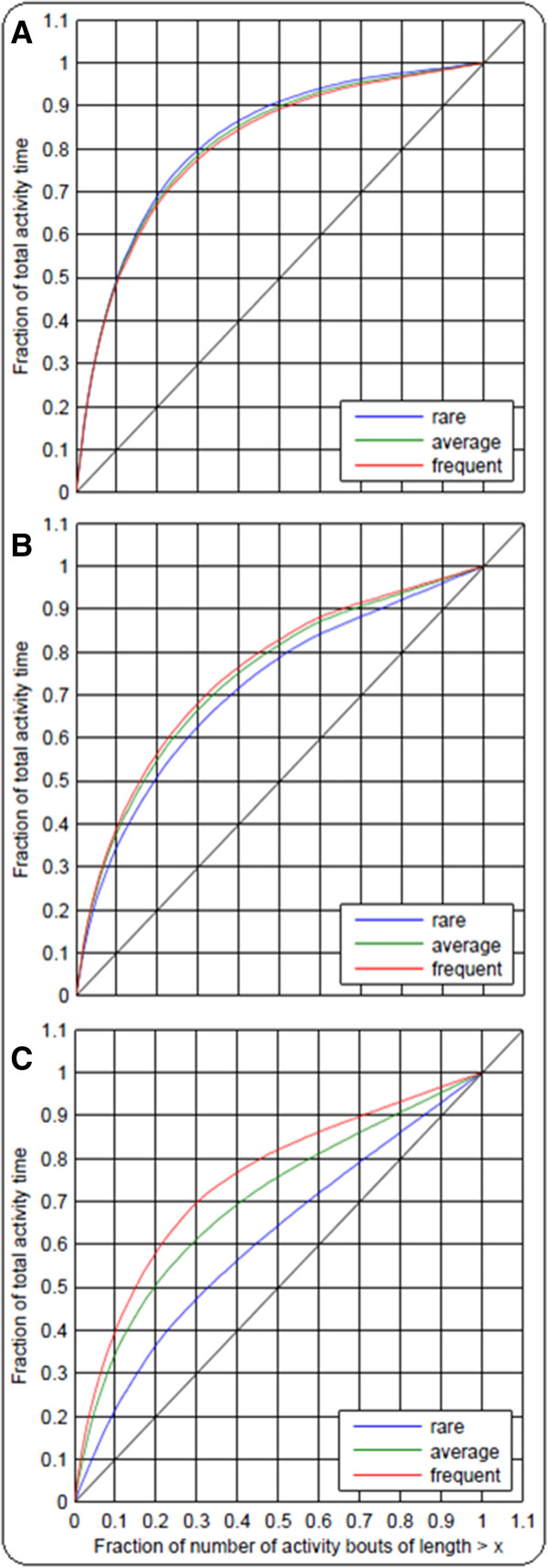
**Lorenz curves of sedentary (A), light (B), and moderate to vigorous (C) physical activity.** The GINI-index (G) corresponds to the area between the curve and the line of perfect equality (G = 0), marked by a solid line. *High G* = mainly few long bouts are responsible for the activity pattern; *low G* = mainly short bouts of similar length contribute to the activity pattern.

Patterns of PA are described in terms of activity bouts. We define a bout as consecutive minutes spent at a specific intensity level without interruption (Figure 2B). Bouts are characterized by their duration and frequency throughout recording time. This is classified by the so-called GINI-index (G), introduced by Chastin and colleagues (Figure 1) [6], which ranges from 0 to 1 and expresses the variability in bout length for a given amount of activity. Examples of high and low G are presented in the additional file (see Additional file 3: Figure S1A-C and Figure S2A-C) with corresponding visualization of PA by means of activity counts, bouts, and Lorenz-curves. A G value close to zero shows a lot of bouts of the same length: in this case, the Lorenz curve converts to the bisecting line. In contrast, a high G value indicates that activity bouts are highly unequal in length. The larger the inequality is, the higher becomes the G value and the larger is the area under the Lorenz curve (Figure 3).

The G value of each intensity level was plotted against the time spent in each intensity level in order to examine associations between the two features. It is important to note that G is a measure of bout distribution, not length. Therefore, further parameters like mean and median length were calculated. Moreover, the percentage of time spent in bouts longer than the median was calculated.

### Subjects characteristics and clinical parameters

Age was divided into four groups: age 65–69, 70–74, 75–79, and over 79. BMI was classified as underweight (< 18.5 kg/m^2^), normal weight (18.5-24.9 kg/m^2^), overweight (25.0-29.9 kg/m^2^) and obese (≥ 30.0 kg/m^2^). No participant was underweight, 25.4% (n = 43) were of normal weight, 51.5% (n = 87) were overweight, and 23.1% (n = 39) were obese. Multimorbidity is the presence of more than one chronic disease of the following 13: hypertension, eye disease, heart disease, diabetes mellitus, joint disease, lung disease, gastrointestinal disease, mental illness, stroke, cancer, kidney disease, neurological disease, and liver disease. Detailed description is available in Kirchberger et al. [14]. Disability was assessed with the Health Assessment Questionnaire Disability Index (HAQ-DI) [15] and was defined as HAQ-DI > 0. For more detailed information see Stobl et al. [16]. Functional exercise capacity was assessed using the six-minute walking test (6MWT) [17] and expressed as six-minute walking distance (6MWD). Lung function was considered as a potential confounder, since the participants of this study were selected based on spirometry values (see Additional file 1, ‘study population’). PA variables (PA times and G values at each intensity level) were tested for association with age, gender, and BMI.

### Statistical analyses

Data was analyzed in SAS 9.2. Differences between the three activity groups (‘rare’, ‘average’, and ‘frequent’) were analyzed using the Kruskal-Wallis test for metric variables and Chi-square test for categorical variables. Wilcoxon’s rank sum test was applied for pairwise comparisons of metric variables. Spearman correlation coefficient was used for correlation analyses. To identify differences regarding individual metric PA variables and categorical subject characteristics, we used Kruskal-Wallis test for three or more groups (BMI and age) and Wilcoxon’s rank sum for two groups (gender). We used Bonferroni correction for all pairwise comparisons.

Collinearity diagnostics were used to test for multicollinearity between explanatory variables. Variables with a tolerance (T = 1 - R^2^) of less than 0.20 were eliminated [18]. A multiple stepwise linear regression model was used to predict exercise capacity. Statistically significant differences were assumed at a significance level of p < 0.05.

## Results

### Subject characteristics and clinical parameters

Table 1 shows the characteristics and clinical parameters of the subjects, stratified by activity group (‘rare’, ‘average’, and ‘frequent’). The final sample comprised 78 men and 91 women, with a median (5%, 95%) age of 73 (65, 86). The distribution of participants over the three different activity groups illustrates that PA decreases with increasing age and BMI. No significant differences were found between men and women.

**Table 1:** Characteristics and clinical parameters of individuals, by activity group

**Characteristic**	**All**	**Rare**	**Average**	**Frequent**	**P value**
	**(n = 168)**	**(n = 42)**	**(n = 84)**	**(n = 42)**	
Age (years)	73.0 (65.0,86.0)	80.5 (66.0,87.0)	72.5 (66.0,84.0)	70.0 (65.0,79.0)	<.0001
Gender, male (%)	46.4 (n = 78)	35.7 (n = 15)	48.8 (n = 41)	52.4 (n = 22)	0.2556
BMI (kg/m^2^)	27.2 (22.6,35.0)	28.8 (23.6,37.6)	27.0 (21.3,34.7)	26.9 (22.8,32.5)	0.0272
Lung function					
FVC (pp.)	102.7 (73.5,128.0)	92.3 (69.1,123.9)	103.2 (77.9,133.2)	112.3 (76.4,127.1)	
FEV_1 (_pp.)	106.4 (65.4,130.8)	85.2 (62.8,130.8)	106.4 (68.2,132.6)	112.5 (67.5,128.3)	
FEV_1_/FVC (L)	0.74 (0.61,0.85)	0.72 (0.61,0.85)	0.74 (0.62,0.86)	0.77 (0.66,0.84)	
Lung group, better (%)	54.4 (n = 92)	38.1 (n = 16)	56.0 (n = 47)	69.1 (n = 29)	0.0164
Multimorbidity, yes (%)	51.8 (n = 87)	69.1 (n = 29)	47.6 (n = 40)	42.9 (n = 18)	0.0312
Disability, yes (%)	41.7 (n = 70)	61.9 (n = 26)	41.7 (n = 35)	21.4 (n = 9)	0.0008
6MWD (m)	466 (274,625)	369 (235,486)	467 (290,593)	536 (379,668)	<.0001

P-values result from Kruskal-Wallis for metric variables and Chi^2^-test for categorical variables. BMI = body mass index, 6MWD = six minute walking distance, pp = percent predicted, L = liter.

Median (5%, 95%).

There were also disparities among the three differently active groups relating to lung function, multimorbidity, disability, and 6MWD. Average PA decreases with decreasing 6MWD, worse lung function, incidence of multimorbidity and incidence of disability. After pairwise comparisons, all significant results remained significant between rarely and frequently active people. In contrast, only disability showed significant results when comparing the frequent with the average group (see Additional file 2: Table S2).

### Characterization of PA

Overall, participants had 8.1 ± 1.5 (mean ± SD) days of valid activity recordings. The mean wear time was 740 ± 114 minutes per day. The average activity per day was 248 cpm. Subjects spent 504 ± 89 minutes in sedentary activities, 252 ± 80 minutes in light activities and 19 ± 21 minutes in MVPA per day according to Freedson and 49 ± 39 minutes in MVPA according to Copeland and Esliger (see Additional file 2: Table S1). Substantial differences can be noted for the time spent in MVPA in all activity groups, with the largest differences in the rarely active group.

**Figure 4:**
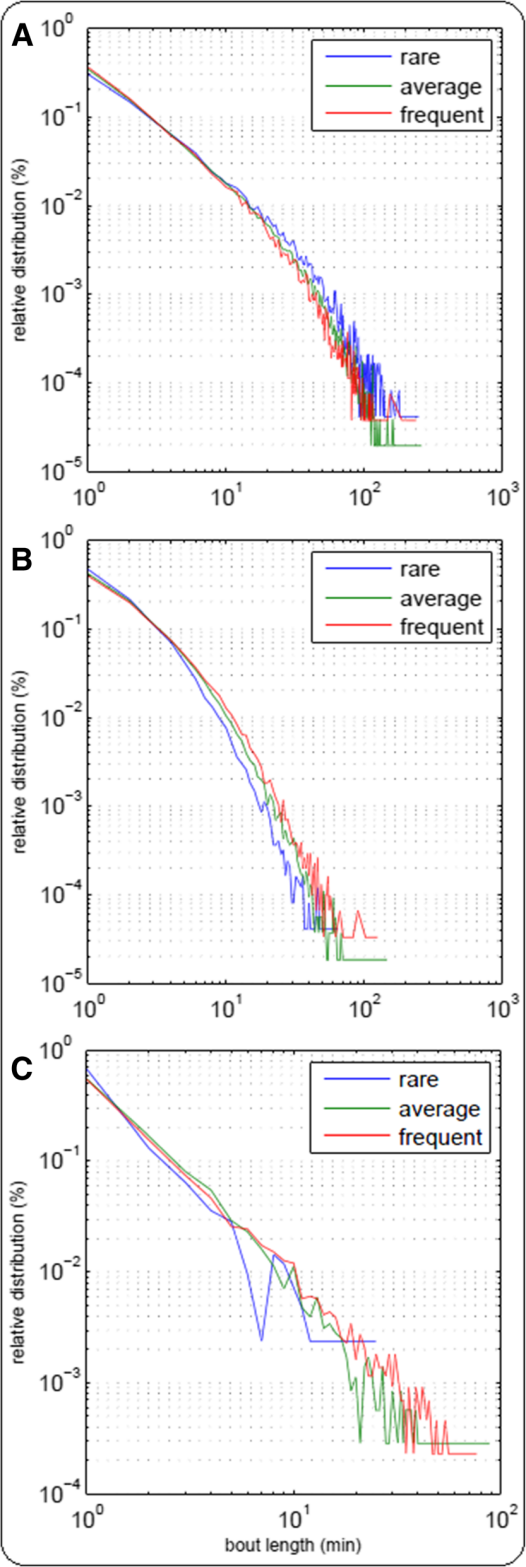
Distribution of PA bouts of sedentary (A), light (B), and moderate to vigorous (C) physical activity.

Table 2 presents the medians for accumulated time spent in PA at different intensity levels in percent (PA time (%)) as well as different variables that describe the patterns of PA in each intensity level. Active people spent proportionally less time in sedentary activity and more time in both the light and MVPA level than less active people. These differences were most obvious at the MVPA level.Percentage of total time spent in bouts greater than the median length, as well as median bout length, show that distribution of bout lengths differs between the activity groups in each intensity level. In the most active group at least 50% of MVPA bouts were shorter than or equal to 1 minute, but bouts longer than this contributed the majority (81%) of total time in MVPA. This trend is attenuated in moderately and rarely active people (70% and 0%). However, the imbalance between the number of bouts and their contribution to accumulation of time in the considered intensity level, here MVPA, can also be observed in the two other intensity levels. Values of the mean bout length support this finding. Since the majority of bouts were short among all intensity levels within all activity groups (Figure 4), median bout lengths differed only slightly, however significantly, between the three groups.

**Table 2:** Values for PA data analysis, by activity group

**Characteristic**	**All**	**Rare**	**Average**	**Frequent**	**P value**
	**(n = 168)**	**(n = 42)**	**(n = 84)**	**(n = 42)**	
**PA (% of time)**					
Sedentary	0.65 (0.50, 0.82)	0.74 (0.66,0.85)	0.65 (0.54,0.74)	0.59 (0.41,0.67)	<.0001
Light	0.32 (0.18,0.48)	0.25 (0.15,0.34)	0.33 (0.22,0.45)	0.35 (0.27,0.54)	<.0001
MVPA	0.02 (0.00,0.08)	0.00 (0.00,0.01)	0.02 (0.00,0.04)	0.05 (0.01, 0.10)	<.0001
**Median BL (min)**					
Sedentary	3.00 (2.00, 4.50)	3.00 (2.00, 5.00)	3.00 (2.00,4.00)	2.00 (2.00, 3.00)	<.0001**
Light	2.00 (1.00, 2.50)	2.00 (1.00, 2.00)	2.00 (2.00, 2.50)	2.00 (2.00, 3.00)	<.0001**
MVPA	1.00 (1.00, 3.00)	1.00 (1.00, 2.00)	1.00 (1.00, 2.50)	1.00 (1.00, 4.00)	0.0256
**% time > median BL**					
Sedentary	0.89 (0.84, 0.92)	0.90 (0.86, 0.92)	0.88 (0.84, 0.92)	0.88 (0.81,0.91)	0.0010
Light	0.75 (0.61, 0.84)	0.71 (0.58, 0.77)	0.76 (0.66, 0.84)	0.78 (0.69, 0.85)	<.0001
MVPA	0.69 (0.00, 0.89)	0.00 (0.00, 0.81)	0.70 (0.00, 0.87)	0.81 (0.48, 0.91)	<.0001
**Mean BL (min)**					
Sedentary	7.08 (4.78, 11.81)	8.64 (6.13, 12.58)	6.91 (4.80, 9.97)	6.31 (4.18, 8.58)	<.0001
Light	3.14 (2.10, 4.76)	2.78 (1.92, 3.44)	3.30 (2.40, 4.65)	3.58 (2.84, 5.45)	<.0001
MVPA	2.21 (1.00, 6.42)	1.00 (1.00, 3.46)	2.26 (1.00, 5.34)	3.70 (1.39, 7.61)	<.0001
**GINI-index**					
G_sedentary_	0.63 (0.57,0.68)	0.65 (0.60, 0.68)	0.63 (0.58,0.68)	0.62(0.57,0.67)	0.0004
G_light_	0.48 (0.37,0.55)	0.44 (0.34,0.48)	0.49 (0.41,0.55)	0.50 (0.44,0.55)	<.0001
G_MVPA_*	0.43 (0.00,0.66)	0.16 (0.00,0.59)	0.44 (0.14,0.66)	0.51 (0.23,0.66)	<.0001

P-values result from Kruskal-Wallis test; PA = physical activity; MVPA = moderate to vigorous PA; BL = bout length; % time > median BL = percentage contribution to the total time in bouts greater than the median bout length; ^*^n = 156 (33/82/41 for rare/average/frequent).**significant p values due to different variances.

Median (5%, 95%).

To demonstrate accumulation of PA at various bout lengths, G (GINI-Index) values and the respective Lorenz curves are presented in Table 2 and Figure 3A-C. G values in all levels must be interpreted in the following way: *high G values*: large difference between min and max bout length and relatively high proportion of long bouts with regard to the overall time; *Low G values:* an activity pattern characterized by a lot of bouts of similar length. *Average G values* may arise in two ways: (i) intermediate difference between min and max bout length or, (ii) considerable differences without favoring short or long bouts. In comparison to high G values, the proportion of long bouts is decreased.

G values differed between the three intensity levels of PA and between the three activity groups within the levels. Values of G tended to decrease with increasing intensity level. At the sedentary level, a high proportion of overall PA time tends to be composed of long bouts (G = 0.63 over all participants), while light PA and MVPA predominately consist of medium bouts (G = 0.48 and G = 0.43 over all participants). Furthermore, frequently active people showed the largest areas under the Lorenz curves and accordingly the highest G values for MVPA and light PA and the lowest G values for sedentary. For rarely active people it was the other way around. Hence, physical exercise was composed of an increased proportion of medium to long bouts in more active compared to less active subjects, whereas sedentary behavior was characterized by a lower proportion of medium to long bouts. Again, the discrepancies were most obvious at the MVPA level.

Associations between PA time spent within an intensity level and the respective G value showed moderate to strong correlations between PA times and PA patterns within intensity levels (r = 0.34 for sedentary, r = 0.65 for light, and r = 0.73 for MVPA).

### Effect of age, gender and BMI on PA intensities

Table 3 presents associations between selected PA variables (PA time and G values within the three intensity levels) and participant characteristics (gender, age group, and BMI group). Except for G for MVPA (G_MVPA_), all PA variables showed significant differences by age. Those over age 79 were more sedentary and less active at all intensities of PA. See Additional file 2: Table S4. Moreover, the G of light PA (G_light_) showed negative associations while that of sedentary PA (G_sedentary_) was positively associated with age, indicating that older subjects tended to spend longer bouts in the sedentary level and shorter bouts in the light level than younger subjects. Gender differences were found only for time in MVPA: men spend more time in MVPA than women. Lastly, obese people showed lower G_MVPA_ values and spent less time in the MVPA level than normal weight persons (see Additional file 2: Table S3b).

**Table 3:** PA variables (intensities and patterns), by gender, age, BMI

**n = 168PA variables**	**Age group**^**1 **^**(p value)**	**Gender (p value)**	**BMI group**^**2 **^**(p value)**
**PA time (%)**			
Sedentary	**<.0001**	0.8300	0.2564
Light	**0.0005**	0.3740	0.6067
MVPA	**<.0001**	**0.0221**	**<.0001**
**GINI-index**			
G_sedentary_	**0.0347**	0.0862	0.3325
G_light_	**0.0115**	0.7577	0.8457
G_MVPA_*	0.2294	0.1038	**0.0003**

^1^age groups: 65–69 years, 70–74 years, 75–79 years, and ≥ 80 years.

^2^BMI groups: normal weight (18.5-24.9 kg/m^2^), overweight (25.0-29.9 kg/m^2^) and obese (≥ 30.0 kg/m^2^).

P-values for comparisons between genders result from Wilcoxon test and for comparisons between Age groups and BMI groups from Kruskal-Wallis test. BMI = body mass index; PA = physical activity; MVPA = moderate to vigorous PA; *n = 156; Significant values are written in bold, p ≤ .05.

### Prediction of FEC

For the prediction of FEC all potential explanatory variables (intensity variables, GINI-indices, individual characteristics and clinical parameters) were included in the initial model. Light PA was set as baseline, and the other two categories were compared to it. As shown in Table 4, the final model explains 56% of the variance in FEC.

**Table 4:** Predictors of functional exercise capacity on multivariate analysis

**Predictor**	**B**	**β**	**P value**	**Partial r**^**2**^	**Model r**^**2**^	**Adjusted r**^**2**^
G_MVPA_	1.0	0.21	<.0001	0.27	0.56	0.54
Age group	−25.0	−0.30	<.0001	0.12		
Gender	−37.2	−0.20	0.0004	0.05		
Lung group	−38.1	−0.20	0.0001	0.05		
Multimorbidity	−32.9	−0.17	0.0005	0.04		
BMI group	−22.1	−0.15	0.0145	0.02		
MVPA	5.7	0.15	0.0169	0.02		

G_MVPA_ = GINI-index of MVPA; MVPA = moderate to vigorous physical activity; B = unstandardized regression coefficient; Beta = standardized regression coefficient; n = 156.

The single biggest predictor of FEC was G_MVPA_ explaining 27% of total variance. 2% more was explained by the total amount of MVPA. Age predicted 12% of variance, and gender, lung group, multimorbidity and BMI together predicted 16%. Younger age, male sex, better lung function, the absence of multimorbidity and lower BMI (at least normal weight) were protective.

## Discussion

Our study shows that the elderly spent 65% of their daily time being sedentary and 35% being active, of which only 2% was MVPA. These results are similar to those reported from other elderly samples [19-21]. Duration of activity bouts decreased with increasing intensity level, i.e. substantially more minutes are continuously spent sedentary than in light activity or MVPA. Other studies which examined the patterns of elderly people in different activity levels are rare. Lord et al. [22] examined the G of sedentary activity and walking in elderly people and presented similar results to ours: sedentary bouts tended to be longer than walking bouts. In contrast, Donaire-Gonalez and colleagues [23] did not find differences concerning the duration of bouts in MVPA and overall PA. However, in their study, the duration of bouts was expressed as the median bout length which may be a less significant measure than the G value itself.

Subjects in the frequently active group were more active than others at all levels of PA, spending more time in both light activity and MVPA than the comparison groups and less time sedentary. PA patterns differed by degree of activity (‘rare’, ‘average’, and ‘frequent’) among all intensity levels. Findings were most clear with regard to the time in MVPA as well as G_MVPA_: the more active a person was, the larger was the proportion of long bouts in light and MVPA and the larger was the proportion of short bouts in the sedentary level.

To our knowledge, the work of Chastin et al. [6] was the only other publication that compared PA patterns of more and less active people. They focused on patterns of sedentary activity and found that the sedentary time of less active subjects was composed of longer rest periods, which is in accordance with our findings.

PA time was correlated with PA patterns: an increased proportion of long bouts (higher G values) was positively correlated with the accumulated PA time in the respective intensity level. This effect was most significant for MVPA. Comparisons between the different activity groups support this finding. Accordingly, a higher proportion of long bouts in light PA as well as in MVPA may be beneficial to increase the overall activity time in rarely active people. In contrast, given a certain activity level, the proportion of long bouts in MVPA must be increased in order to increase the overall activity level and related health benefits [24], as patterns of light PA do not differ significantly between the average and frequent group (see Additional file 2: Table S2).

However, these recommendations may need to be adjusted for each individual, particularly for people in the rare group. Exercise capacity and specific health conditions [2,25] may limit the ability to perform PA. In our sample, rarely active people typically had reduced FEC, poor lung function, higher prevalence of multimorbidity, and higher prevalence of disability. However, many disabled or multimorbid persons in our sample achieved higher levels of PA than people without disability or multimorbidity. Thus, disability and multimorbidity do not necessarily limit PA.

We tested associations between PA times and G values with age, gender, and BMI within each intensity level, in order to examine whether and how particular intensity levels and patterns differ in specific groups. Older participants, obese persons and women seem to be particularly prone to a sedentary lifestyle. The reduced average activity of obese subjects is particularly associated with decreased time in MVPA, whereas the reduced average activity of elderly participants is due to a decreased time in both light PA and MVPA.

Examinations of the relationship between PA and gender are inconsistent. Gardner and colleagues [7], for example, demonstrated that women with intermittent claudication aged 65 ambulate slower than men. Jakicic et al. [26] objectively measured the MVPA patterns of 59-year-old overweight and obese individuals with type 2 diabetes mellitus, and found that men have a larger amount of bouts ≥10 minutes in that level than women. Those findings agree with ours, whereas their results of BMI and age are contrary: they found no associations between MVPA bouts and age or BMI [26]. In line with our results, other studies found that the proportion of long bouts (8–10 min/day) of MVPA declines with increasing BMI and advancing age [27,28]. Lastly, a study with subjects aged 70–88 identified younger age and lower BMI as significant predictors of walking. There was no correlation between PA and gender [22].

Younger age, lower BMI, male sex, better lung function, absence of multimorbidity, more time and longer bouts (higher G values) in the MVPA level, emerged as significant predictors of exercise capacity: they explained 56% of the total variance in FEC. It is important to note that G_MVPA_ accounted for 27% of the variance, by far the largest single predictor. This finding indicates that in addition to the well-known relationship between FEC and PA in terms of duration and intensity, there is also one in terms of patterns. Correlations between FEC and PA characteristics (such as intensities or patterns) have been examined and evidenced before [29-31]. However, these studies either used univariate analysis or failed to consider detailed information about PA (like intensities and patterns). Hernandes et al. [29], for example, demonstrated that the intensity of movement correlates with 6MWD in healthy elderly individuals (r = 0.49; p < 0.01) and that walking time is positively associated with FEC in COPD patients (r = 0.42; p < 0.01). However, no information about further predictors of FEC was shown. Moreover, no advice about the level of intensity is given.

Our findings underline the current PA guidelines for older adults [2,25], which imply that activity should be at least moderate intensity. Moreover, our results support the fact that a higher proportion of longer bouts predict FEC better than a higher proportion of shorter bouts, with potential greater effect on health [31].

The present study is the first one that examined PA patterns in terms of G among three different intensity levels (sedentary PA, light PA, and MVPA) and thus presents detailed and differentiated information about activity patterns of different intensities in elderly people. We identified associations between times and patterns of PA in different intensity levels and examined the relationship regarding FEC. However, due to the cross-sectional study design the direction of causality of the examined associations cannot be assured.

Recognized limitations of accelerometers include their inability to detect non-walking activity such as resistance training or cycling. Thus they are likely to underestimate such activities [19] and related bout lengths. Another limitation of this study is the questionable validity of the cut-points applied to classify activities into intensity levels. No general standard for transforming activity data into different intensity levels exists [32] although there are many validation and calibration studies. We chose the algorithm by Freedson et al. [10] because it is the most often used validated algorithm for ActiGraph sensors [11] and therefore has the highest potential to provide comparable data. Since the calibration study of Copeland and Esliger [13] was been performed specifically with older adults, we also present PA variables based on these cut points in order to increase the comparability of this novel method for the future. As expected, differences for time spent in MVPA are detectable, particularly among individuals who are infrequently active. Direct comparison of PA variables would enable systematic measurement of how choice of cut-points influences prediction of PA levels and patterns. We consider this a very interesting scientific issue and plan to discuss it in a separate paper.

In conclusion, both time spent in MVPA and G_MVPA_ emerged as important predictors for functional exercise capacity. Time in MVPA can most profitably be increased by increasing the proportion of long bouts which enhances activity levels and meets recommendations for PA while simultaneously increasing G. These recommendations can be followed by most older adults, but those with functional or health-related limitations may need to adjust accordingly. In rarely active people (commonly characterized by higher age, higher BMI, reduced functional exercise capacity, worse lung function, and multimorbidity and disability) even light activity, i.e. a higher proportion of long bouts in light PA, may increase average activity levels.

## Competing interests

None of the authors has any conflicts of interest to disclose. The authors are responsible for the content and the writing of this paper. The KORA research platform (KORA, Cooperative Health Research in the Region of Augsburg) was initiated and financed by the Helmholtz Zentrum München, German Research Center for Environmental Health (formerly GSF, National Research Center for Environment and Health), which is funded by the German Federal Ministry of Education and Research and by the State of Bavaria. KORA Age was financed by the German Federal Ministry of Education and Research (BMBF FKZ 01ET0713). Further support was provided by the BMBF funded Competence Network ASCONET, subnetwork COSYCONET (FKZ 01GI0882). The research was supported by the Graduate School of Information Science in Health (GSISH) and the Technische Universität München Graduate School. Lukas Gorzelniak received university grant monies as a PhD scholarship from the 3/1/2009 to 6/30/2012. André Dias is supported by the Portuguese Foundation for Science and Technology (FCT), by scholarship SFRH/BD/39867/2007 and Research Council of Norway Grant No. 174934.

## Authors’ contributions

SO wrote the manuscript. SO, AD, AH, and HS were responsible for data analysis and interpretation as well as statistical expertise. LG, AD, DN, SK, AP, KK, AH and HS were involved in data acquisition as well as in concept and design. All authors were involved in manuscript revision. All authors read and approved the final manuscript.

## Authors’ information

Alexander Horsch and Holger Schulz shared last authorship.

## Supplementary Material

Additional file 1 Methods. Click here for file

Additional file 2: Table S1 Comparison of cut-points: Freedson vs. Copeland, by activity group. Median (5%/95%). **Table S2.** Pairwise comparisons of characteristics and clinical parameters, by activity group. **Table S3.** Pairwise comparisons of PA variables, by activity group. **Table S4.** Pairwise comparisons of PA variables, by age group and BMI group. Click here for file

Additional file 3**Illustration of physical activity counts, bouts and Lorenz-curves for two subjects with different GINI-Indices. Figure S1A-C – subject I:** Visualization of physical activity (1 day) of a subject with a relatively high GINI-Index for moderate to vigorous physical activity (GMVPA=0.72). **A) Activity counts of a day provided in 4 segments:** The two lines (at 100 and 1952 counts) reflect the cut-points for light activity and moderate to vigorous physical activity (MVPA). Values ≤ 100 correspond to sedentary PA, values between 100-1951 to light PA and ≥ 1952 to MVPA. **B) Corresponding bouts of the day provided in 4 segments:** A bout is defined as consecutive minutes spent in a specific intensity level, i.e. sedentary, light or MVPA, without an interruption. The intensity levels are provided for 1-minute epochs ( 1-minute). **C) Lorenz-Curves:** The GINI-index (G) corresponds to the area between the curve and the line of perfect equality (G = 0), marked by a solid line. The figure shows a G_MVPA_ = 0.72, which means that mainly few long bouts are responsible for the activity pattern. **Figure S2A-C – subject II:** Visualization of physical activity (1 day) of a subject with a relatively low GINI-Index for moderate to vigorous physical activity (G_MVPA_=0.10). **A) Activity counts of a day provided in 4 segments:** The two lines (at 100 and 1952 counts) reflect the cut-points for light activity and moderate to vigorous physical activity (MVPA). Values ≤ 100 correspond to sedentary PA, values between 100-1951 to light PA and ≥ 1952 to MVPA. **B) Corresponding bouts of the day provided in 4 segments:** A bout is defined as consecutive minutes spent in a specific intensity level, i.e. sedentary, light or MVPA, without an interruption. The intensity levels are provided for 1-minute epochs (  1-minute). **C) Lorenz-Curves:** The GINI-index (G) corresponds to the area between the curve and the line of perfect equality (G = 0), marked by a solid line. The figure shows a G_MVPA_=0.10, which means that mainly short bouts of similar length contribute to the activity pattern.Click here for file
