# Supplementary material for: Exploring patterns of accelerometry-assessed physical activity in elderly people
Source: Int J Behav Nutr Phys Act. 2014 Feb 28;11:28. doi: 10.1186/1479-5868-11-28 (PMC4016218; doi:10.1186/1479-5868-11-28)
Supplement: Additional file 1 — Methods. [file 1479-5868-11-28-S1.doc]

**Additional File 1**

**Methods**

**Study population**

The participants were a subsample of the ‘Kooperative Gesundheitsforschung in der Region Augsburg’ (KORA) study. The KORA-Age study investigates the determinants and consequences of health status changes of older adults in a representative population based sample over a period of 3 years. Details have been described previously . Since lung function is a strong predictor for overall morbidity and mortality , a subsample of two hundred elderly participants, without diagnosis of bronchitis, asthma, emphysema, chronic obstructive pulmonary disease (COPD) or a combination of these diseases, was selected for the present examination. Based on extreme spirometry values the subjects were extracted from the first and fourth quartiles of the KORA-Age study population and grouped into a ‘better’ or ‘worse’ lung function group. The intention of the selection criteria was to examine the relationship between lung function and numerous health outcomes. However, that was not the focus of the present study; here, lung function was considered as a potential confounder. Nine subjects refused the attendance due to personal or organizational reasons.

The KORA-Age study was approved by the ethics committee of the State Board of Physicians, written informed consent has been obtained from the participants and all investigations have been conducted according to the principles expressed in the Declaration of Helsinki.

**Data collection**

PA levels from the non-dominant side of the hip were assessed by means of a GT3X (ActiGraph, Pensacola, FL, USA) accelerometer in 191 elderly healthy subjects from the KORA-Age cohort during everyday life. In the case of contra indications, participants were able to change the side on which the sensor was worn. To avoid sensor damages, participants were instructed to take them off for water activities (e. g. swimming, fishing). The devices were initialized to collect data from all three axes at a 2-second epoch time and a 30Hz data sample frequency in order to enable a recording of at least 11 days. By initializing more than a single axis, the VMU (the vectoral sum of activity in the three orthogonal directions) representation was automatically stored as well. PA was recorded up to 10 days, starting from the day at the study center where the accelerometers were handed out, until the eleventh day, which was the day the subjects were instructed to return the devices.

**Wear time calculation**

The PA data were downloaded using the ActiLife Software 4.0 (ActiGraph) and were further processed using MATLAB R2012a (MathWorks, Natick, MA, USA), a high-level data analysis environment. We applied an adjusted algorithm of Hecht et al. to the cohort data, based on the triaxial VMU representation to determine the wearing time. PA data from the first recorded day as well as any other day with a wear time of less than 10 hours were eliminated, accordant to the recommendations for objective devices . Furthermore, subjects were excluded if they did not reach a minimum of four valid days . According to the stated criteria, 168 subjects were found to be eligible for analysis (88.9%). In total, 23 subjects were excluded, 19 due to insufficient days of recordings, and four due to accelerometer malfunctions (e.g. broken battery).

**Data processing and accelerometer measures**

The PA data recorded in 2 seconds epochs was converted into the well-established 60 second epochs using MATLAB. For the evaluation of PA, uniaxial data (vertical axis) were used: cut off levels for the different intensity levels were well established for uniaxial recordings, and comparability of the vertical axis of the GT3X with previous ActiGraph devices is proven . Based on the uniaxial, 60s epochs several variables were obtained from accelerometer data to represent the characteristics of PA. Figure 1 presents an overview of the most important PA variables concerning this analysis. Parameters used in this study were:

Overall PA and average activity

Overall PA involves all activity counts of the vertical axis measured during the whole recording time. Average PA is defined as the mean number of activity counts per minute (cpm) and expresses the total number of registered counts for all valid days divided by wearing time. We used this variable to describe associations between average PA and subject characteristics (age, gender, BMI) as well as clinical parameters (lung function, 6MWD). The distribution of this variable was divided into 3 categories: one group included subjects in the lowest quartile (‘rare’: < 25th percentile), a second group those in the two intermediate quartiles (‘average’: ≥ 25th – < 75th percentile) and a third group contained participants in the highest quartile (‘frequent’: ≥ 75th percentile).

Intensity levels

Activity counts were assigned to the different intensity levels using cut points published by Freedson et al. for light, moderate, and vigorous PA (figure 2A). Since the often-applied Freedson kcal equation provides most comparable data and gives close estimates of both light and moderate PA , this classification was the method of choice. In order to compare Freedsons’ cut points with the more recently developed cut points by Copeland and Esliger for elderly people , we present the results based on both equations in the supplement (see supplement, table 2). Sedentary behavior was classified as fewer than 100 counts per minute as explored by different authors and applied in several studies . Due to the lack of time that older people spend in vigorous activities, moderate and vigorous PA were combined into one group and defined as MVPA.

PA Patterns

Patterns of PA can be described in different ways, e.g. by means of activity bouts. In the present study, a bout is defined as consecutive minutes spent in a specific intensity level, i.e. sedentary, light or MVPA, without an interruption (figure 2B).To determine the activity pattern of a subject, activity bouts are characterized by their duration (bout length) and frequency (amount of bouts) throughout the whole recording time. This information is grouped together by the so-called GINI-index (G), introduced by Chastin and colleagues (figure 1) . The index illustrates how the activity time in a specific intensity level is accumulated with respect to the bout lengths. The index value, G value, ranges from 0 and 1. For the visualization of G Lorenz curves were used (figure 3). A G value close to zero shows that a lot of bouts of the same length contribute to the pattern of accumulation. In this case, the Lorenz curve converts to the bisecting line. In contrast, a high G value indicates that bouts of unequal lengths are responsible for the activity pattern. The larger the inequality is, the higher becomes the G value and the larger is the area under the Lorenz curve. The G value of each intensity level was plotted against the PA time spent in each intensity level in order to examine associations between the two features. It is important to note that G provides relative values and does not give information about the dimensions of the bouts. Therefore, further parameters like mean and median bout length were calculated. Moreover, the percentage of time spent in bouts longer than the median bout length was calculated.

Two examples of a relatively high and low G for MVPA (G_MVPA_) with corresponding visualization of PA by means of activity counts, bouts, and Lorenz-curves are presented in the supplement (Figure S1A-C and Figure S2A-C).

**Subjects characteristics and clinical parameters**

As described above (see ‘overall PA and average activity’) associations between average PA and subject characteristics (age, gender, BMI) were examined. In order to gain information about the relationship between PA and the characteristics within each intensity level, different PA variables (PA times and G values of each intensity level) were tested in terms of associations with age, gender, and BMI. To assess age dependency, the subjects were divided into four age groups (65-69 y, 70-74 y, 75-79 y, and ≥ 80 y). Due to a lack of subjects in high ages, persons aged 80 years and older were merged into one group. In line with the WHO classifications, BMI was classified into underweight (< 18.5 kg/m^2^), normal weight (18.5-24.9 kg/m^2^), overweight (25.0-29.9 kg/m^2^) and obese (≥ 30.0 kg/m^2^). Multimorbidity was defined as the presence of ≥ 2 chronic diseases out of a list of 13 chronic diseases (hypertension, eye disease, heart disease, diabetes mellitus, joint disease, lung disease, gastrointestinal disease, mental disease, stroke, cancer, kidney disease, neurological disease, liver disease). A detailed description is available in Kirchberger et al. . Disability was assessed with the Health Assessment Questionnaire Disability Index (HAQ-DI) and was defined as HAQ-DI > 0. Disability registers all impairments, activity limitations and participation restrictions that are often reflected by restrictions in activities of daily living and mobility. For more detailed information regarding disability within the KORA-Age framework see Stobl et al. . Functional exercise capacity was assessed using the six minute walking test (6MWT) and expressed as six minute walking distance (6MWD). Lung function was considered as a potential confounder, since the participants of this study were selected based on spirometry values (see ‘study population’).

**References**

1. Peters A, Döring A, Ladwig KH, Meisinger C, Linkohr B, Autenrieth C, al. e: **Multimorbidity and successful aging: the population-based KORA-Age study.** *Z Gerontol Geriatr* 2011, **44:**41-54.

2. Pellegrino R, Viegi G, Brusasco V, Crapo RO, Burgos F, Casaburi R, Coates A, van der Grinten CP, Gustafsson P, Hankinson J, et al: **Interpretative strategies for lung function tests.** *Eur Respir J* 2005, **26:**948-968.

3. Hecht A, Ma S, Porszasz J, Casaburi R, for the COPD Clinical Research Network: **Methodology for Using Long-Term Accelerometry Monitoring to Describe Daily Activity Patterns in COPD.** *COPD* 2009, **6:**126-129.

4. Ward DS, Evenson KR, Vaughn A, Rodgers AB, Troiano RP: **Accelerometer Use in Physical Activity: Best Practices and Research Recommendations.** *Med Sci Sports Exerc* 2005, **37:**582-588.

5. Matthews CE, Hagströmer M, Pober DM, Bowles HR: **Best Practices for Using Physical Activity Monitors in Population Based Research - review.** *Med Sci Sports Exerc* 2012, **44:**68–76.

6. Trost S, Mciver KL, Pate RR: **Conducting Accelerometer-Based Activity. Assessments in Field-Based Research.** *Med Sci Sports Exerc* 2005, **37:**531-543.

7. Pruitt LA, Glynn NW, King AC, Guralnik JM, Aiken EK, Miller G, Haskell WL: **Use of accelerometry to measure physical activity in older adults at risk for mobility disability.** *J Aging Phys Act* 2008, **16:**416-434.

8. Sasaki JE, John D, Freedson PS: **Validation and comparison of ActiGraph activity monitors.**  *J Sci Med Sport.*2011, **14:**411-416.

9. Kaminsky LA, Ozemek C: **A comparison of the Actigraph GT1M and GT3X accelerometers under standardized and free-living conditions.** *Physiological Measurement* 2012, **33:**1869-1876.

10. Freedson PS, Melanson E, Sirard J: **Calibration of the Computer Science and Applications, Inc. accelerometer.** *Med Sci Sports Exerc* 1998, **30:**777-781.

11. Bento: **Use of accelerometry to measure physical activity in adults and the elderly.** *Rev Saúde Pública* 2012, **46:**561-570.

12. Crouter SE, Clowers KG, Bassett DR: **A novel method for using accelerometer data to predict energy expenditure.** *J Appl Physiol* 2006, **100:**1324-1331.

13. Copeland J, Esliger D: **Accelerometer Assessment of Physical Activity in Active, Healthy Older Adults.** *Journal of Aging and Physical Activity* 2009, **17:**17-30.

14. Freedson PS, Pober D, Janz KF: **Calibration of Accelerometer Output for Children.** *Med Sci Sports Exerc* 2005, **37:**523-530.

15. Matthews CE, Chen KY, Freedson PS, Buchowski MS, Beech BM, Pate RR, Troiano RP: **Amount of time spent in sedentary behaviors in the United States, 2003-2004.** *Am J Epidemiol* 2008, **167:**875-881.

16. Treuth MS, Schmitz K, Catellier DJ, McMurray RG, Murray DM, Almeida MJ, Going S, Norman JE, Pate R: **Defining Accelerometer Thresholds for Activity Intensities in Adolescent Girls.** *Med Sci Sports Exerc* 2004**:**1259-1266.

17. Mattocks C, Leary S, Ness A, Deere K, Saunders J, Tilling K, Kirkby J, Blair SN, Riddoch C: **Calibration of an accelerometer during free-living activities in children.** *Int J Pediatr Obes* 2007, **2:**218-226.

18. Evenson KR, Buchner DM, Morland KB: **Objective Measurement of Physical Activity and Sedentary Behavior Among US Adults Aged 60 Years or Older.** *Prev Chronic Dis.* 2011, 9:E26.

19. Chastin SFM, Granat MH: **Methods for objective measure, quantification and analysis of sedentary behaviour and inactivity.** *Gait & Posture* 2010, **31:**82–86.

20. Kirchberger I, Meisinger C, Heier M, Zimmermann A, Thorand B, Autenrieth C, Peters A, Ladwig K-H, Döring A: **Patterns of Multimorbidity in the Aged Population. Results from the KORA-Age Study.** *PLoS ONE* 2012, **7:**e30556.

21. Fries J, Spitz P, Young D: **The dimensions of health outcomes: the health assessment questionnaire, disability and pain scales. .** *J Rheumatol* 1982, **9:**789–793.

22. Strobl R, Müller M, Emeny R, Peters A, Grill E: **Distribution and determinants of functioning and disability in aged adults - results from the German KORA-Age study.** *BMC Public Health* 2013, **13:**137.

23. **ATS statement: guidelines for the six-minute walk test.** In *Am J Respir Crit Care Med*, 2002, **166:**111-117.
