# Supplementary material for: Exploring patterns of accelerometry-assessed physical activity in elderly people
Source: Int J Behav Nutr Phys Act. 2014 Feb 28;11:28. doi: 10.1186/1479-5868-11-28 (PMC4016218; doi:10.1186/1479-5868-11-28)
Supplement: Additional file 2: Table S1 — Comparison of cut-points: Freedson vs. Copeland, by activity group. Median (5%/95%). Table S2. Pairwise comparisons of characteristics and clinical parameters, by activity group. Table S3. Pairwise comparisons of PA variables, by activity group. Table S4. Pairwise comparisons of PA variables, by age group and BMI group. [file 1479-5868-11-28-S2.doc]

**Additional File 2**

Table 1 – Comparison of cut-points: Freedson vs. Copeland, by activity group. Median (5%/95%)

|  | all | rare | average | frequent |
| --- | --- | --- | --- | --- |
|  | (n = 168) | (n = 42) | (n = 84) | (n = 42) |
| **MVPA**  **(% of time)** |  |  |  |  |
| Freedson | 0.02 (0.00/0.08) | 0.00 (0.00/0.01) | 0.02 (0.00/0.04) | 0.05 (0.01/0.10) |
| Copeland | 0.05 (0.01/0.16) | 0.02 (0.00/0.03) | 0.05 (0.02/0.09) | 0.11 (0.06/0.21) |
| **MVPA Time**  **(minutes/ day)** |  |  |  |  |
| Freedson | 11.6 (0.2/64.0) | 1.0 (0.1/7.3) | 13.3 (0.9/34.4) | 39.9 (7.8/81.1) |
| Copeland | 41.7 (4.1/118.3) | 11.8 (1.5/25.0) | 41.7 (12.4/66.9) | 90.1 (52.0/156.9) |

PA = physical activity; MVPA = moderate to vigorous physical activity;

Cut-point for MVPA by Freedson = 1952 and by Copeland = 1041;

In the following MVPA time (minutes per day) is presented in mean (SD) values in order to compare it with other authors who presented their results by means (SD):

Freedson: 18.6 (20.9)

Copeland: 48.9 (39.4)

Table 2 - Pairwise comparisons of characteristics and clinical parameters, by activity group.

| Characteristic | rare vs. frequent | average vs. frequent |
| --- | --- | --- |
|  | p-value | p-value |
| Age (years) | **>.0001** | 0.0415 |
| Gender, male (%) | 0.1239 | 0.7055 |
| BMI (kg/m^2^) | **0.0091** | 0.7464 |
| Lung function better (%) | **0.0045** | 0.1567 |
| Multimorbidity, yes (%) | **0.0156** | 0.6132 |
| Disability, yes (%) | **0.0002** | **0.0247** |

P-values result from Wilcoxon tests and Chi^2^-tests. Bonferroni correction was used for pairwise comparisons. Accordingly the p-value was 0.05/2=0.025. Significant values are written in bold. PA= physical activity; MVPA=moderate to vigorous PA; BL=bout length; %time > median BL=percentage contribution to the total time in bouts greater than the median bout length

Table 3 – Pairwise comparisons of PA variables, by activity group.

| PA variable | rare vs. frequent | average vs. frequent |
| --- | --- | --- |
|  | p-value | p-value |
| **PA (% of time)** |  |  |
| Sedentary | **<.0001** | **<.0001** |
| Light | **<.0001** | **0.0107** |
| MVPA | **<.0001** | **<.0001** |
| **Median BL (min)** |  |  |
| Sedentary | **<.0001** | 0.0218 |
| Light | **<.0001** | 0.1682 |
| MVPA | **0.0123** | 0.4197 |
| **%time > median BL** |  |  |
| Sedentary | **0.0005** | 0.2899 |
| Light | **<.0001** | 0.0359 |
| MVPA | **<.0001** | **0.0009** |
| **Mean BL (min)** |  |  |
| Sedentary | **<.0001** | 0.0182 |
| Light | **<.0001** | 0.0174 |
| MVPA | **<.0001** | **0.0030** |
| **GINI-index** |  |  |
| G_sedentary_ | **0.0002** | 0.0669 |
| G_light_ | **<.0001** | 0.0222 |
| G_MVPA_* | **<.0001** | **0.0082** |

P-values result from Wilcoxon tests with Bonferroni correction for pairwise comparisons. Accordingly the p-value was 0.05/3=0.0167. Significant values are written in bold. PA= physical activity; MVPA=moderate to vigorous PA; BL=bout length; %time > median BL=percentage contribution to the total time in bouts greater than the median bout length

Table 4: Pairwise comparisons of PA variables, by age group and BMI group

| Characteristic | 70-74 y^1^  p-value | < 75-79 y^1^  p-value | ≥80 y^1^  p-value | overweight^2^  p-value | obese^2^  p-value |
| --- | --- | --- | --- | --- | --- |
| **PA (% of time)** |  |  |  |  |  |
| Sedentary | 0.5837 | 0.0498 | **<.0001** | 0.5054 | 0.1245 |
| Light | 0.6284 | 0.0517 | **0.0003** | 0.3434 | 0.4467 |
| MVPA | 0.5565 | 0.1089 | **<.0001** | 0.7458 | **<.0001** |
| **GINI-index** |  |  |  |  |  |
| G_sedentary_ | 0.2855 | 0.0905 | **0.0067** | 0.2326 | 1.0000 |
| G_light_ | 0.6513 | 0.1932 | **0.0079** | 0.5667 | 0.6844 |
| G_MVPA_* | 0.5047 | 0.1373 | 0.0703 | 0.7700 | **0.0009** |

^1^ reference group: < 70 y; ^2^reference group: normal weight

P-values result from Wilcoxon tests with Bonferroni correction for pairwise comparisons. Accordingly the p-value was 0.05/3=0.0167 for age and 0.05/2=0.025 for BMI. Significant values are written in bold. PA= physical activity; MVPA=moderate to vigorous PA; BL=bout length; %time > median BL=percentage contribution to the total time in bouts greater than the median bout length
