# Supplementary figures and images for: Exploring patterns of accelerometry-assessed physical activity in elderly people
Source: Int J Behav Nutr Phys Act. 2014 Feb 28;11:28. doi: 10.1186/1479-5868-11-28 (PMC4016218; doi:10.1186/1479-5868-11-28)

Figure S1A-C

A

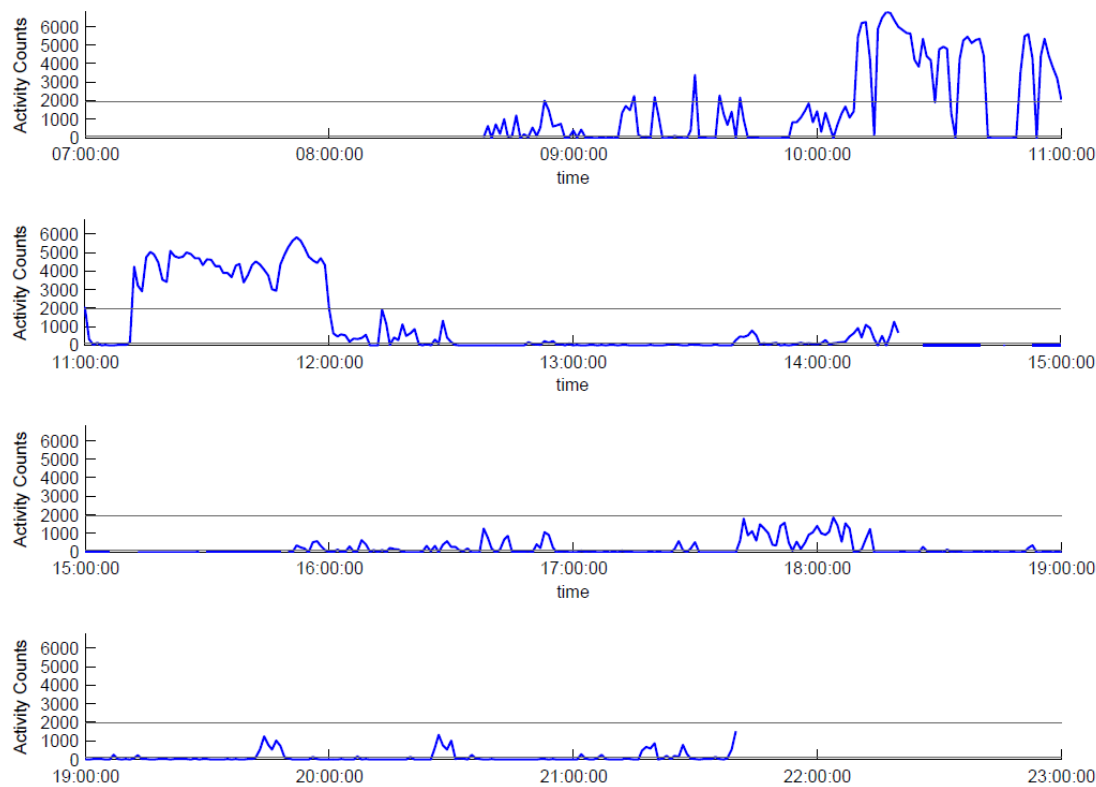

B

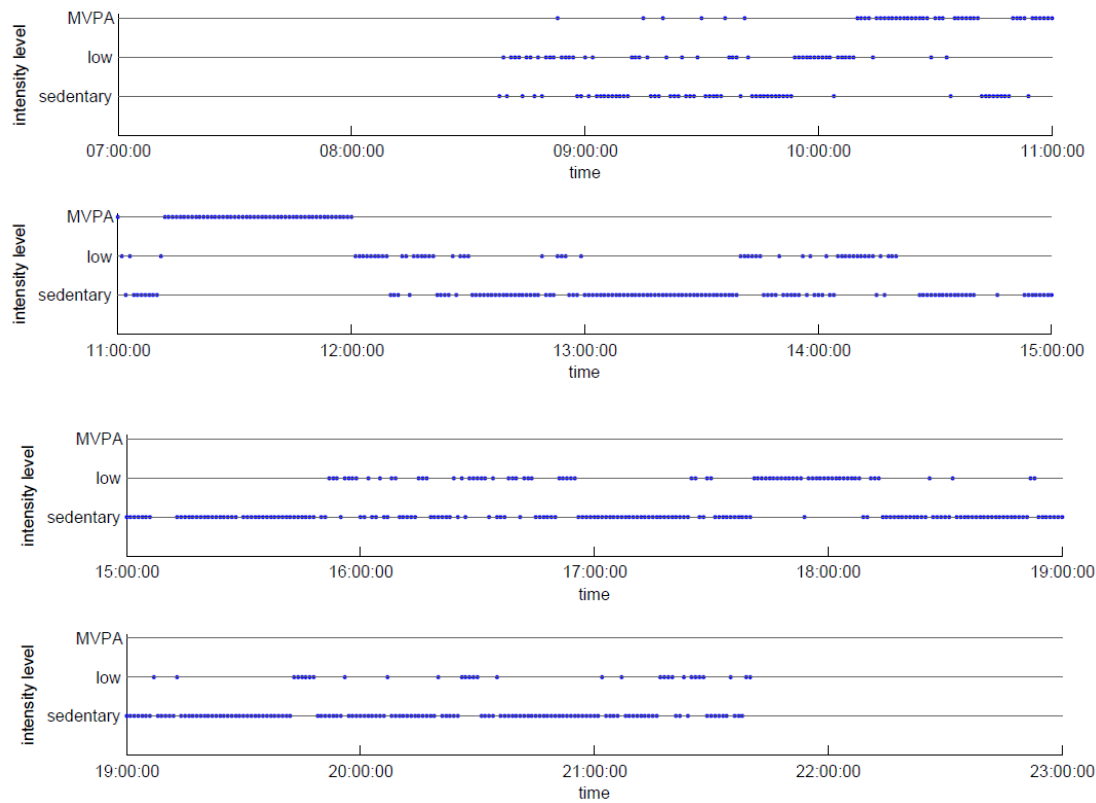

C

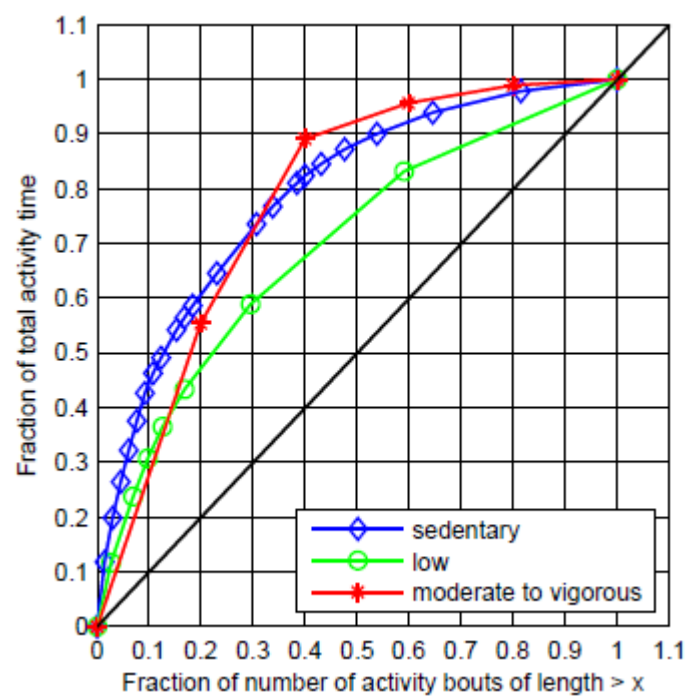

Figure S2A-C

A

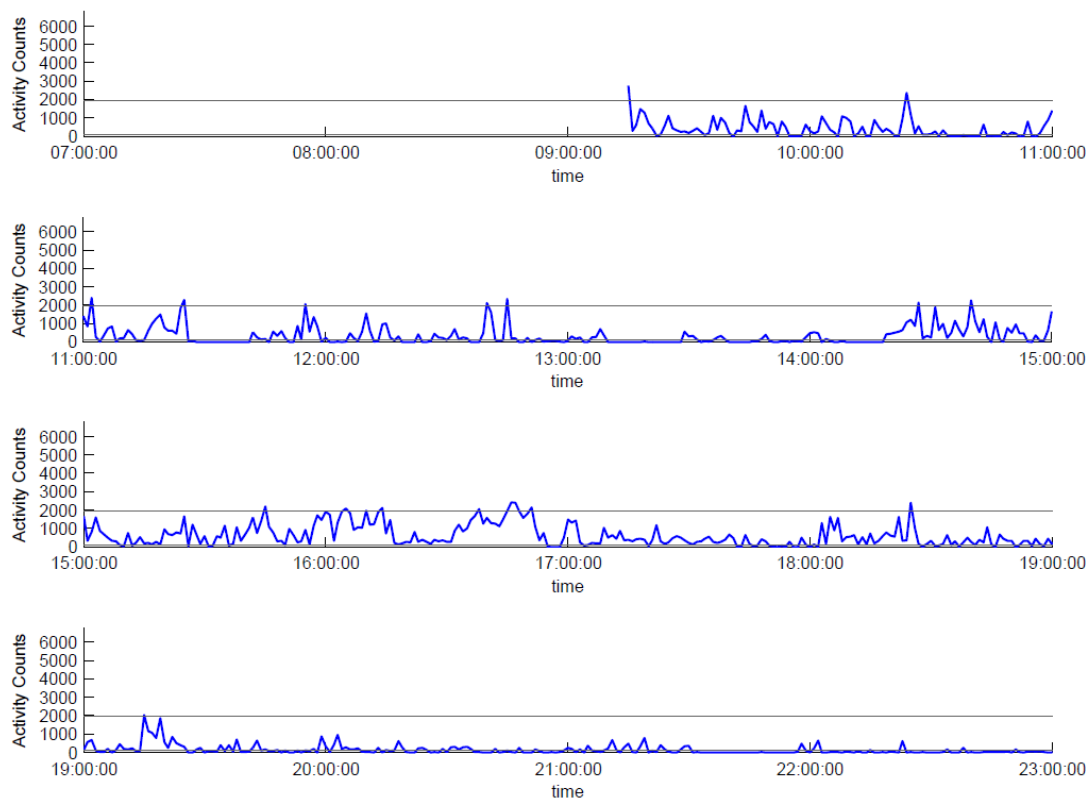

B

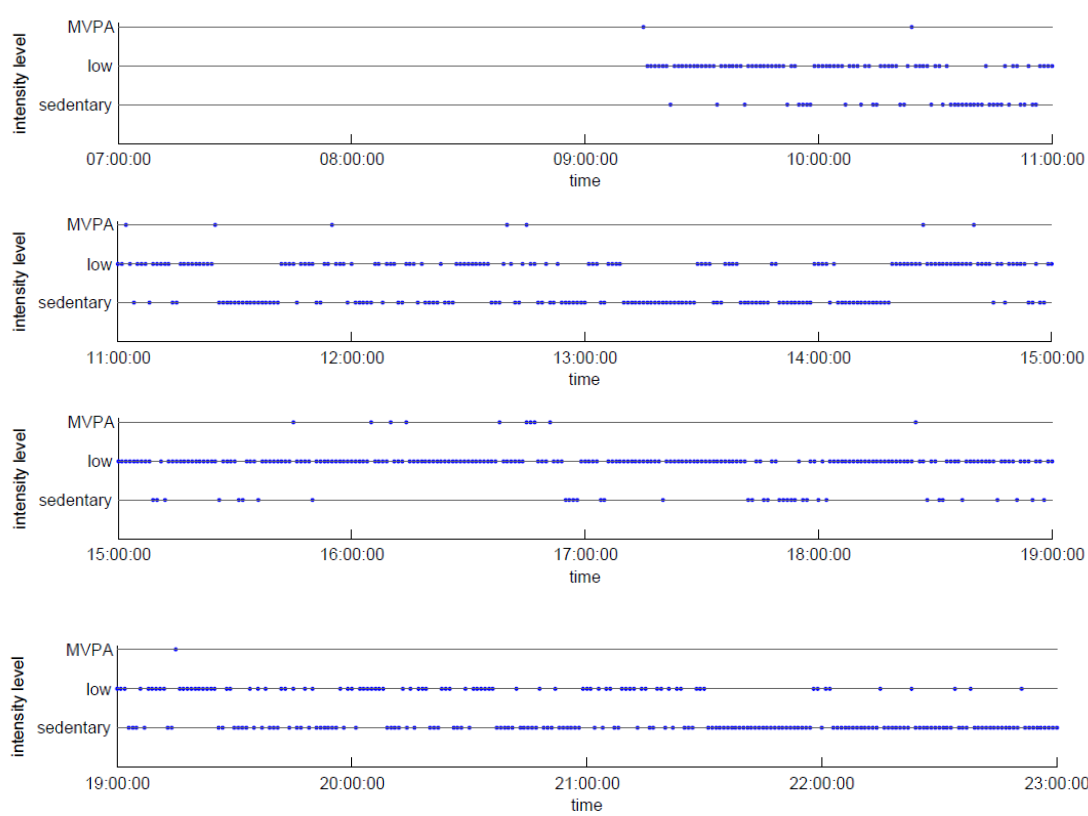

C

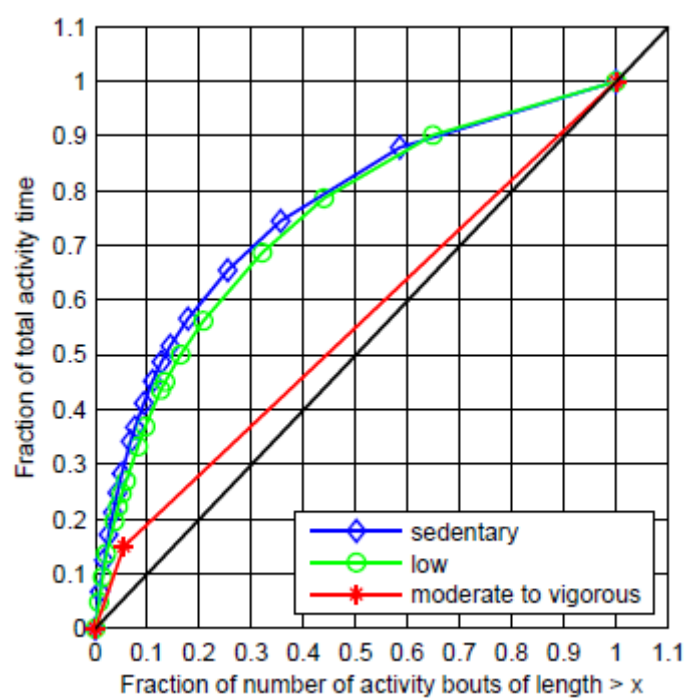

Supplement: Additional file 3 — Illustration of physical activity counts, bouts and Lorenz-curves for two subjects with different GINI-Indices. Figure S1A-C – subject I: Visualization of physical activity (1 day) of a subject with a relatively high GINI-Index for moderate to vigorous physical activity (GMVPA=0.72). A) Activity counts of a day provided in 4 segments: The two lines (at 100 and 1952 counts) reflect the cut-points for light activity and moderate to vigorous physical activity (MVPA). Values ≤ 100 correspond to sedentary PA, values between 100-1951 to light PA and ≥ 1952 to MVPA. B) Corresponding bouts of the day provided in 4 segments: A bout is defined as consecutive minutes spent in a specific intensity level, i.e. sedentary, light or MVPA, without an interruption. The intensity levels are provided for 1-minute epochs ( 1-minute). C) Lorenz-Curves: The GINI-index (G) corresponds to the area between the curve and the line of perfect equality (G = 0), marked by a solid line. The figure shows a GMVPA = 0.72, which means that mainly few long bouts are responsible for the activity pattern. Figure S2A-C – subject II: Visualization of physical activity (1 day) of a subject with a relatively low GINI-Index for moderate to vigorous physical activity (GMVPA=0.10). A) Activity counts of a day provided in 4 segments: The two lines (at 100 and 1952 counts) reflect the cut-points for light activity and moderate to vigorous physical activity (MVPA). Values ≤ 100 correspond to sedentary PA, values between 100-1951 to light PA and ≥ 1952 to MVPA. B) Corresponding bouts of the day provided in 4 segments: A bout is defined as consecutive minutes spent in a specific intensity level, i.e. sedentary, light or MVPA, without an interruption. The intensity levels are provided for 1-minute epochs ( 1-minute). C) Lorenz-Curves: The GINI-index (G) corresponds to the area between the curve and the line of perfect equality (G = 0), marked by a solid line. The figure shows a GMVPA=0.10, which means that mainly sho [file 1479-5868-11-28-S3.pdf]
